# Algal–Bacterial Symbiotic Systems for Resource Recovery and High-Value Product Generation from Ammonia-Rich Food Wastewater: A Review

**DOI:** 10.3390/foods15213893

**Published:** 2026-10-30

**Authors:** Meng Ma, Ran Li, Bing Guo, Zichao Zhao, Tao Sun, Xinhao Gao, Li Yao

**Affiliations:** State Key Laboratory of Nutrient Use and Management, Key Laboratory of Wastes Matrix Utilization, Ministry of Agriculture and Rural Affairs, Institute of Agricultural Resources and Environment, Shandong Academy of Agricultural Sciences, Jinan 250100, China; 15001956061@163.com (M.M.); lran0507@163.com (R.L.); guo321bing@163.com (B.G.); 2008zzc@163.com (Z.Z.); suntao1007@126.com (T.S.); sdnkygxh@163.com (X.G.)

**Keywords:** algal–bacterial consortium, food wastewater, ammonia nitrogen removal, quorum sensing, resource recovery, high-value products

## Abstract

The global food processing industry generates substantial volumes of wastewater characterized by elevated ammonia nitrogen concentrations, presenting both significant environmental challenges and opportunities for nutrient recovery. Conventional biological treatment consumes substantial energy for aeration and sludge handling. Algal–bacterial symbiosis represents a promising circular-economy strategy that combines wastewater purification with biomass production. This review synthesizes current knowledge on the application of algal–bacterial consortia for treating ammonia-rich food wastewater, with emphasis on the synergistic mechanisms underpinning nitrogen removal, biomass production, and the generation of high-value products. It examines microalgae–bacteria interactions—mediated by oxygen–carbon dioxide exchange, nutrient conversion, extracellular polymeric substance dynamics, and quorum sensing signaling. Among these mechanisms, quorum sensing acts as one of several key regulatory pathways, modulating microbial community succession, biofilm formation, metabolic pathway expression, and stress tolerance, thereby contributing to pollutant removal efficiency and biomass resource quality, together with gas exchange, nutrient assimilation, and microbial metabolism. The review also evaluates recent advances in reactor configurations that facilitate scalable implementation. Furthermore, it discusses the valorization of harvested biomass into aquafeed, biofertilizers, biofuels, and bioactive compounds, alongside the principal barriers to commercialization. Finally, it highlights emerging research directions involving synthetic biology, artificial intelligence-assisted process control, and a biorefinery that may accelerate the transition of algal–bacterial symbiosis systems from laboratory research toward practical food wastewater treatment and resource recovery applications.

## 1. Introduction

The global food processing industry is one of the largest consumers of freshwater and producers of wastewater, generating effluents rich in organic matter, nitrogen, and phosphorus [1,2]. Dairy processing, starch production, brewing, soybean processing, aquaculture, and fruit/vegetable processing collectively discharge vast quantities of wastewater containing ammonia nitrogen concentrations that can range from tens to hundreds of milligrams per liter [3]. For example, soy sauce production generates approximately 6–9 tons of wastewater per ton of product, with Chinese soy sauce companies producing roughly 50 million tons of wastewater annually [4]. The discharge of such untreated or inadequately treated effluents into aquatic environments promotes eutrophication, oxygen depletion, and disruption of ecological balance [5]. Conventional treatment approaches, including activated sludge, physicochemical methods, and membrane filtration, are effective at pollutant removal but are energy-intensive, generate substantial sludge, and fail to recover the valuable nutrients embedded in the waste stream [2,6]. The traditional nitrification–denitrification process faces challenges including high energy consumption and environmental unsustainability [7]. Physical and chemical methods, although effective, are expensive and may cause secondary environmental pollution [8]. These limitations have motivated the search for alternative treatment technologies that combine environmental effectiveness with economic viability and resource recovery potential.

In response to these challenges, microalgae-based wastewater treatment has gained renewed attention for its ability to couple contaminant removal with biomass generation and CO_2_ sequestration [9]. Among the various microalgal configurations, algal–bacterial symbiotic systems have emerged as particularly promising platforms [10]. These consortia leverage the complementary metabolic activities of photosynthetic microalgae and heterotrophic bacteria: microalgae supply oxygen through photosynthesis, supporting bacterial oxidation of organic matter and ammonia, while bacteria release carbon dioxide that fuels algal growth [11]. This mutualistic relationship creates a self-sustaining treatment system that reduces external aeration requirements and simultaneously produces harvestable biomass enriched in proteins, lipids, carbohydrates, and bioactive compounds.

Food wastewater presents a compelling case for algal–bacterial treatment due to its high nutrient content and the potential for biomass valorization within food supply chains [12]. The ammonia-rich nature of many food processing effluents provides an ideal nitrogen source for microalgal growth, while the organic carbon content supports bacterial metabolism [2]. Recent studies have demonstrated that algal–bacterial consortia can achieve ammonia nitrogen removal efficiencies exceeding 90% in food wastewater treatment, while the harvested biomass offers opportunities for production of aquafeed, biofertilizers, biofuels, and bioactive extracts [13]. Despite these advances, several interconnected scientific, technological, and economic challenges continue to hinder large-scale implementation. The high cost of cultivation and downstream processing, variability in biomass quality, concerns regarding contaminant accumulation in wastewater-grown biomass, and limited understanding of the complex microbial interactions within consortia all pose significant barriers [14]. Moreover, while numerous studies have examined microalgae-based treatment of municipal or agricultural wastewater, systematic reviews focusing specifically on algal–bacterial symbiosis system for ammonia-rich food wastewater are comparatively scarce.

**Figure 1:**
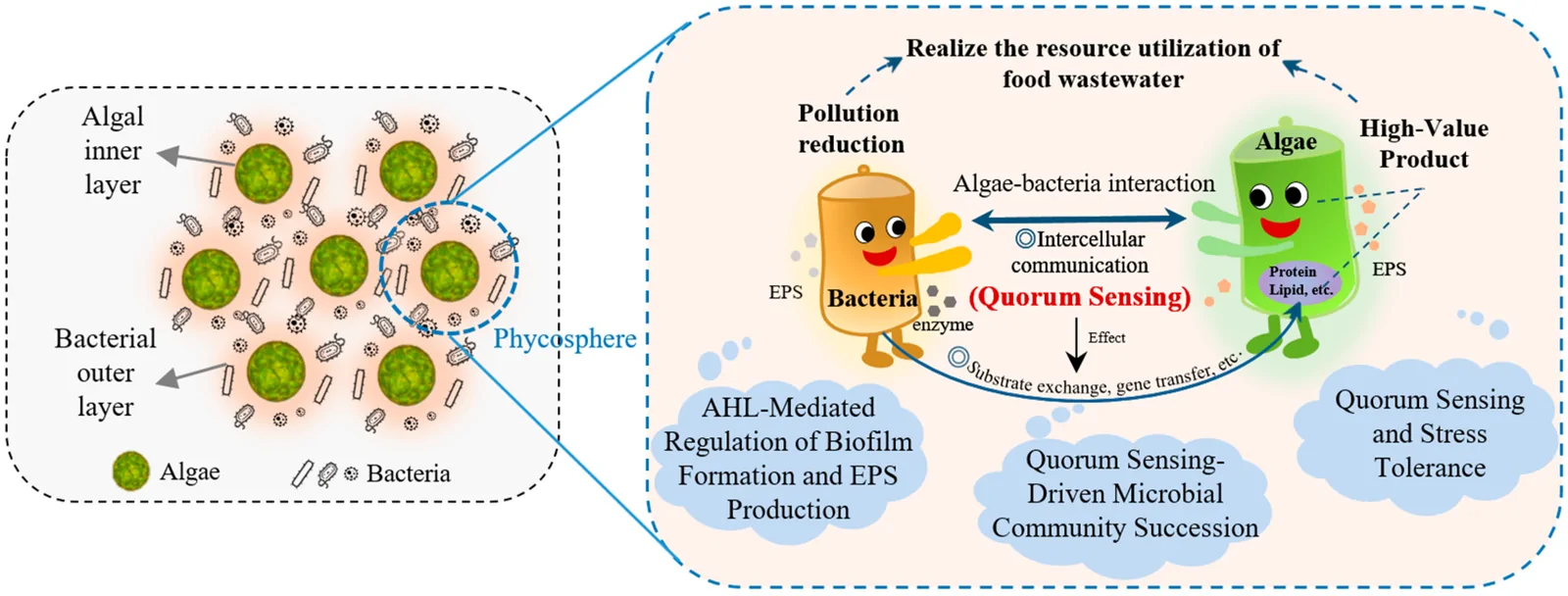
Comprehensive analysis of the different functional roles of algal–bacterial symbiotic systems in food wastewater treatment and resource recovery.

Previous reviews have addressed algal–bacterial systems for wastewater treatment from different perspectives. Sirohi et al. reviewed waste mitigation and resource recovery from food industry wastewater using microalgae–bacterial consortia, covering dairy, starch, and aquaculture effluents [2]. Anand et al. provided a broad overview of algae–bacteria consortia for wastewater decontamination and production of biodiesel, bioethanol, biohydrogen, biofertilizers, and animal feed [13]. Ren et al. discussed the development and challenges of algal–bacterial symbiosis systems, including formation mechanisms and biotechnological applications [10]. In contrast, this review focuses specifically on ammonia-rich food wastewater and treats ammonia nitrogen as the central pollutant. It further provides a systematic analysis of quorum sensing—covering AHL molecular basis, cross-kingdom signal perception, QS–EPS–biofilm feedback, community succession, and stress tolerance—and links ammonia assimilation to the generation of high-value products. This review aims to fill this gap by providing a comprehensive synthesis of the diverse functional roles of algal–bacterial symbiotic systems within food wastewater treatment and resource recovery (Figure 1). It integrates biological, engineering, and perspectives to illustrate how these consortia can be strategically incorporated into wastewater treatment systems. This review integrates mechanistic understanding—particularly the interplay among gas exchange, nutrient cross-feeding, EPS dynamics, and quorum sensing—with reactor engineering and product valorization, and evaluates the technical, economic, and operational challenges. Finally, it proposes future research directions involving emerging technologies to accelerate the transition of algal–bacterial symbiosis system from laboratory research toward practical food wastewater treatment and resource recovery applications.

## 2. Characteristics of Food Wastewater and Treatment Challenges

### 2.1. Ammonia-Rich Food Wastewater: Characteristics and Treatment Challenges

Food processing wastewaters exhibit considerable diversity in composition depending on the source industry. Dairy wastewater is characterized by high concentrations of organic matter (COD: 2000–20,000 mg L^−1^) and nitrogen (TN: 50–800 mg L^−1^) [2,15]. Soy sauce wastewater, generated from microbial fermentation using soybeans, wheat, and other raw materials, contains significant organic loads and is frequently treated by biological methods due to its excellent biodegradability [16]. Despite this diversity, a unifying characteristic across these waste streams is the presence of elevated ammonia nitrogen, which arises from protein degradation during food processing, cleaning operations, and metabolic excretion. This ammonia-rich nature is the defining feature of the food waste streams considered in this review, as it both challenges conventional treatment and creates opportunities for nutrient recovery through algal–bacterial symbiotic systems [6,17].

### 2.2. Limitations of Conventional Treatment Approaches

Conventional biological treatment of food wastewater relies on activated sludge processes, achieving effective organic matter and nitrogen removal through nitrification–denitrification. However, these systems are energy-intensive, requiring substantial aeration to support aerobic bacterial metabolism [18]. The high ammonia concentrations common in food waste streams can inhibit nitrifying bacteria, leading to process instability and incomplete nitrogen removal. Furthermore, activated sludge systems generate large quantities of waste sludge with limited reuse potential, perpetuating the linear disposal paradigm [19,20].

Physical and chemical treatment methods, including coagulation, flocculation, membrane filtration, and electrochemical treatment, can achieve high removal efficiencies but are constrained by high operational costs and chemical consumption [21]. Eliminating nitrogen and phosphorus is relatively challenging and costly, and certain chemical agents can cause secondary pollution of water bodies [22]. These limitations have motivated the search for alternative treatment technologies that combine environmental effectiveness with economic viability and resource recovery potential.

## 3. Algal–Bacterial Symbiotic Systems: Principles and Mechanisms

### 3.1. Fundamentals of Algal–Bacterial Symbiosis

Algal–bacterial symbiotic systems are founded on the complementary metabolic activities of photosynthetic microalgae and heterotrophic bacteria [11]. In these consortia, microalgae generate oxygen through photosynthesis, which aerobic bacteria utilize to degrade pollutants in the wastewater; CO_2_ and inorganic substances released during degradation in turn support the growth and metabolism of microalgae [23,24]. This reciprocal gas exchange creates a self-regulating system that can sustain treatment performance with reduced or eliminated external aeration [25,26].

Beyond gas exchange, algae and bacteria engage in more complex metabolic interactions. Microalgae release extracellular polymeric substances (EPS), dissolved organic carbon, and growth-promoting metabolites such as vitamins and amino acids that support bacterial growth. In turn, bacteria mineralize complex organic substrates into simpler compounds—including inorganic nitrogen and phosphorus—that are readily assimilated by algae [27]. This metabolic cross-feeding enhances overall nutrient removal efficiency and creates a more resilient microbial ecosystem [28,29]. This microbial ecosystem, which is the microscale area around algal cells, is called the phycosphere [30]. The phycosphere is a nutrient-rich microenvironment that supports dense bacterial communities and mediates extensive metabolic exchange. This bidirectional metabolic cooperation, together with quorum sensing (QS) signaling, extracellular enzyme activity, and chemically induced gene expression, produces a complex exometabolome that reprograms gene expression in all partners [29,30].

### 3.2. Quorum Sensing as One of Several Regulatory Mechanisms in Algal–Bacterial Symbiosis

#### 3.2.1. Molecular Basis of Quorum Sensing

**Figure 2:**
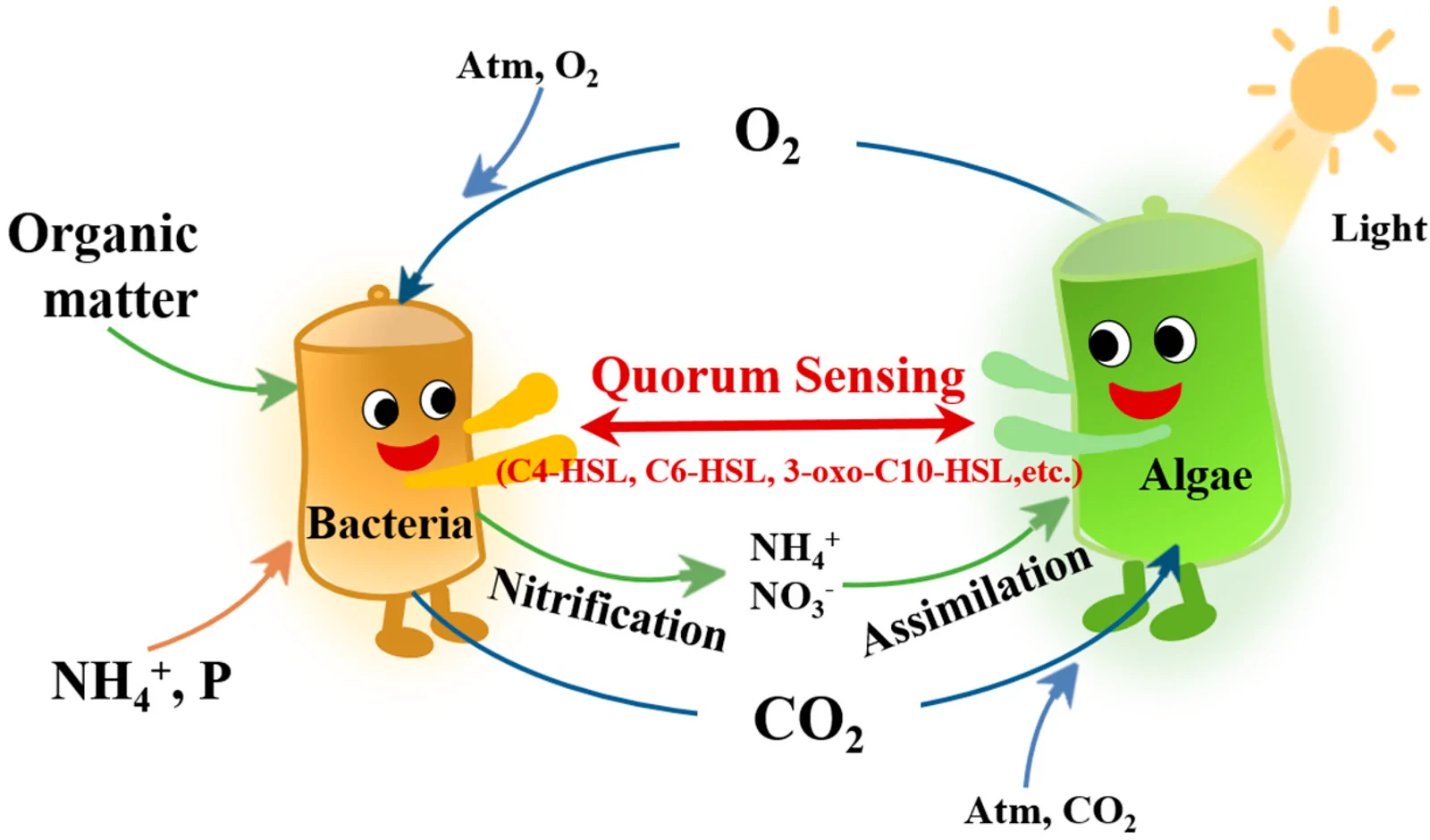
The quorum sensing system between microalgae and bacteria.

QS is the process by which bacteria release signaling molecules such as auto-inducers and acyl-homoserine lactones (AHLs) to communicate with others in the consortia [31,32,33]. In algal–bacterial symbiosis system, QS represents a sophisticated cell-to-cell communication mechanism that enables coordinated population-level behaviors in response to cell density (Figure 2) [34].

N-acyl-homoserine lactones are the primary QS signal molecules in Gram-negative bacteria and have been most extensively studied in algal–bacterial symbiosis system. Different bacteria secrete AHLs with varying side chain lengths (e.g., C4-HSL, C6-HSL, 3-oxo-C10-HSL), each with distinct biological activities and regulatory targets [35,36]. As bacterial population density increases, AHL concentrations accumulate to threshold levels, activating specific gene expression programs [37].

Microalgae have been shown to respond to specific bacterial QS signals, particularly AHLs. Zhou et al. provided the first direct experimental evidence that eukaryotic microorganisms can sense and respond to prokaryotic QS molecules. In their study, model microalga *Chlorophyta* sp. self-aggregated into 200 μm bioflocs by secreting 460–1000 kDa aromatic proteins upon exposure to AHLs extracted from activated sludge bacteria, achieving a settling efficiency of up to 41%, whereas cells remained freely suspended in the absence of AHLs. Transcriptome analysis further revealed that this metabolic response was underpinned by the up-regulation of aromatic protein synthesis pathways from tyrosine and phenylalanine, assisted by anthranilate accumulation. This demonstrates that microalgae not only perceive the presence of bacterial QS signals but also reprogram their metabolic pathways accordingly, thereby establishing a molecular basis for cross-kingdom communication in algal–bacterial consortia [38]. AHLs have been shown to regulate photosynthesis in *Chlorella sorokiniana*, promoting biomass and lipid accumulation. For instance, C8-HSL and C14-HSL increased the biomass of *C. sorokiniana* by 7.42% and 13.86% and lipid content by 11.31% and 21.87%, respectively, accompanied by up to 28.81% enhancement in the maximum photosynthetic rate [39]. Furthermore, C8-HSL enhanced cell density by 30.9% and neutral lipid content by 33.1% through ROS-mediated metabolic reprogramming, with coordinated upregulation of genes involved in photosynthesis, acetyl-CoA generation, and fatty acid biosynthesis [40]. From this, it can be seen that the impact of QS molecules secreted by bacteria promoting microalgae growth on microalgae is increasingly being documented. Despite these observations, the receptors for AHLs in microalgae have not been identified, and the downstream signal transduction pathways remain largely unknown. Most reported responses—including aromatic protein synthesis, lipid accumulation, and ROS-mediated metabolic reprogramming—have been documented in a limited number of species, and their generality across different algal–bacterial pairs is not established.

#### 3.2.2. AHL-Mediated Regulation of Biofilm Formation and EPS Production

AHL-mediated QS contributes to microbial communication and regulation within algal–bacterial biofilms, in conjunction with EPS production and nutrient exchange [41]. AHLs have shown positive effects on the production of aromatic proteins in EPS during bioflocculation [42]. The QS-EPS-biofilm axis constitutes a positive feedback loop: QS signals upregulate EPS synthesis genes, promoting polysaccharide and protein secretion [42]; EPS serves as the “cement” of biofilms, enhancing microbial attachment and aggregation; and biofilm formation increases local cell density, further amplifying QS signal accumulation and response [43,44].

The variations in AHLs provided direct evidence for more frequent interspecies communication, facilitating the ecological reconfiguration in the biofilm. Specifically, in an algal–bacterial biofilm system achieving over 93% total nitrogen removal without external aeration, AHL-mediated QS enhanced microbial communication and regulation, as evidenced by co-occurrence network analysis revealing increased negative correlations (from 36.07% to 39.38%) and reduced community vulnerability (from 0.037 to 0.028), collectively contributing to biofilm stability and resilience [7]. This ecological reconfiguration was driven by the selective enrichment of signal-responsive bacterial communities whose relative abundance was positively correlated with nitrogen and phosphorus removal performance [35].

EPS are critical interfacial mediators in algal–bacterial symbiosis system. EPS is primarily composed of polysaccharides, proteins, humic substances, and nucleic acids, co-secreted by microalgae and bacteria. EPS serves multiple functions: (1) as a structural scaffold maintaining floc/biofilm stability; (2) providing binding sites (functional groups such as –OH and –CH_3_) for nutrient and pollutant adsorption and transport; (3) serving as carbon/energy reserves utilized by microorganisms during nutrient limitation; and (4) protecting microorganisms from oxidative stress, heavy metal toxicity, and other environmental pressures [45,46,47,48,49,50].

Notably, AHL-mediated QS also positively regulates bacterial aggregation and EPS production, with aggregated communities exhibiting enhanced EPS secretion and altered EPS composition ratios that affect biofilm formation [51]. Su et al. reported that the addition of AHLs augmented polysaccharide secretion but had little effect on protein secretion in a filamentous algal–bacterial symbiotic system, indicating that AHLs alter the composition and content of EPS in a component-selective manner [52]. C12-HSL was identified as a key signal molecule mediating communication between Chlorella and Gram-negative bacteria, and its exogenous addition enhanced flocculation activity and aromatic protein production, while quorum-sensing inhibitor vanillin suppressed this effect, confirming the AHL-specific regulatory role [42]. Semi-continuously operated microalgal–bacterial biofilms demonstrate that soluble EPS cross-feeding and bound EPS nitrogen and phosphorus turnover lead to improved pollutant removal. Under non-axenic conditions, enhanced cross-feeding of soluble EPS reduced residual nitrogen, phosphorus, and total organic carbon by 70.1%, 50.3%, and 32.3%, respectively, compared to axenic conditions, with nitrogen and phosphorus turnover from loosely bound to tightly bound EPS reaching 2.0–7.2 and 2.5–5.5 folds [53]. Functional genera and enhanced EPS drive synergistic pollutant degradation. Synergistic interactions via elevated EPS production and carbohydrate metabolism genes enhance oxidative stress resistance and stability [54]. At the molecular level, AHLs dynamically regulate EPS biosynthetic pathways. Under C8-HSL stimulation, the biosynthetic modules of most hydrophobic amino acids (e.g., Trp, Phe, Leu, Val, Cys, and Ile) were upregulated, while the relative abundance of genes involved in the biosynthesis of hydrophilic amino acids (e.g., Arg, Glu, Gln, and Lys) decreased, rendering EPS more hydrophobic and thereby enhancing microbial aggregation ability and granular structural stability [55]. While QS-enhanced EPS production stabilizes biofilms, excessive EPS secretion may increase mass transfer resistance and complicate harvesting. In membrane-based systems, QS-mediated EPS accumulation could exacerbate membrane fouling, although this has not been systematically evaluated in algal–bacterial food wastewater treatment.

#### 3.2.3. Quorum Sensing-Driven Microbial Community Succession

AHL-mediated QS is involved in microbial community succession and metabolic pathway regulation in algal–bacterial biofilm systems, interacting with other factors such as oxygen gradients, nutrient availability, and EPS dynamics. Within algal–bacterial symbiotic biofilms, the dominant genera identified include *Candidatus Brocadia* (anaerobic ammonium-oxidizing bacteria, AnAOB), Nitrosomonas (ammonia-oxidizing bacteria, AOB), and Geitlerinema (microalgae), with relative abundances of 13.86%, 6.37%, and 2.88%, respectively. Compared with earlier stages, the abundance of genes related to nitrogen metabolism pathways (anaerobic ammonium oxidation, denitrification, and ammonia assimilation) increased, indicating enhanced nitrogen transformation capacity in the algal–bacterial symbiotic stage [7]. Sun et al. constructed an algal biofilm reactor and achieved an improvement in nitrogen removal efficiency under low C/N ratios, with algal assimilation contributing approximately 50% of nitrogen removal. The introduction of algae increases the enrichment of bacteria related to denitrification and algal growth and enhances the stability of community structure [56]. QS-driven community succession enables the system to adapt to changing environmental conditions and maintain functional stability. Research has found that QS genes (such as yidC and secG) of cyanobacteria in mature algal–bacterial aerobic granular sludge (ABGS) are significantly upregulated, while genes related to biofilm formation (such as rpoS, glgC, and cysE) are also upregulated. The study clearly indicates that microbial aggregates such as algae particles and sludge rely on tightly coordinated microbial interactions to maintain structural stability and functional performance [57]. Another study found that enhanced QS improved the fitness of rapidly growing bacteria in sludge and biofilm communities, significantly increasing the production of extracellular substances (proteins and polysaccharides). Network analysis shows that enhanced QS significantly promotes ecological interactions between taxa, especially collaborative interactions [58]. QS-bacteria drove changes in community diversity and connectivity, with biofilm formation playing a critical role in this process. The co-occurrence network metrics reported in these studies describe community structure but do not establish causal relationships between QS and treatment performance. Direct evidence linking specific AHL molecules to changes in the metabolic activity of defined receiver populations is still lacking.

#### 3.2.4. Quorum Sensing and Stress Tolerance

QS plays a critical role in the adaptation responses of algal–bacterial consortia to environmental stresses. QS bacteria-driven community changes enhance system resilience under adverse conditions, including high ammonia concentrations, salinity stress, and contaminant exposure. The oxidative stress generated by the metabolism of ammonia oxidizing bacteria (AOB) induces the activation of prophages in lysogenic bacteria, triggering cell lysis and releasing intracellular components, followed by upregulation of QS and promotion of EPS biosynthesis and secretion. Transcriptome and proteomic analysis showed that the presence of *N. europaea* significantly upregulated QS- and EPS-related genes in *E. coli* (λ+) by 2.14–2.93-fold and 2.81–3.11 fold, respectively. Surviving bacteria exhibit enhanced prophage bacterial symbiotic relationships and activated toxin antitoxin systems, enhancing their resilience to environmental stress [59]. Zhou et al. systematically investigated the role of AHL driven QS in enhancing the resistance of microbial biofilms to chloramphenicol stress, demonstrating that AHL-mediated QS can positively regulate the physicochemical properties of biofilms, thereby enhancing their resistance to pollutant stress [60]. By promoting up-regulation of gene expression in bio-augmentation bacteria, QS enhances their colonization rates and improves contaminant degradation within treatment systems.

The adaptive responses of algal–bacterial consortia are characterized by increased bacterial diversity and connectivity, with QS bacteria-driven community changes and biofilm formation playing a critical role [45]. This QS-mediated stress tolerance is particularly relevant for food wastewater treatment, where high ammonia concentrations and variable loading conditions pose significant challenges to system stability. Under the condition of a total nitrogen content of up to 1000 mg L^−1^ in the influent, the dense aggregates induced by diatomaceous earth enhanced the QS activity mediated by AHL (increased total AHL concentration, enriched QS related genes) while enhancing the electron transfer ability, reducing the total nitrogen content in the effluent to 5.10 ± 0.68 mg L^−1^ and improving the TN removal efficiency by 12.95% [61].

### 3.3. Nitrogen Removal Pathways

Nitrogen removal in algal–bacterial symbiosis system is a coordinated process involving multiple pathways rather than a single biological mechanism. The main mechanisms underlying nitrogen removal in these systems are nitrification–denitrification and assimilation in the form of microbial protein. The contributions of different denitrification pathways were quantified by parallel operation of bacterial aerobic granular sludge (BAGS) and ABGS reactors and selective inhibition of nitrification using allyl thiourea. Both systems achieve > 99% ammonia removal, but nitrification/denitrification contributes about 99% in BAGS and only about 53% in ABGS, with the rest being undertaken by assimilation pathways. When nitrification is inhibited, BAGS only retains < 2% of its original ammonia removal capacity, while ABGS still maintains about 33% of its removal capacity, confirming the substantial contribution of assimilation pathways in algal bacterial systems [62].

Algal assimilation: Microalgae directly assimilate ammonium nitrogen (NH_4_^+^–N) into intracellular amino acids, proteins, and other cellular constituents, converting dissolved inorganic nitrogen into recoverable biomass. Kong et al. found that the mechanism for nitrogen removal is mainly the assimilation of microalgae or microalgae bacterial communities during cultivation [63]. At the level of total nitrogen removal, algal assimilation contributes 28.1–40.8%, while bacterial denitrification contributes 2.9–26.5%, clearly confirming that algal assimilation is the primary pathway for total nitrogen removal [64].

Nitrification: Ammonia-oxidizing bacteria, including Nitrosomonadaceae in the microalgae–bacteria consortium, are responsible for the nitrification process. Autotrophic bacteria can convert ammonia through nitrification, where ammonia is oxidized to nitrite and nitrate by two types of bacteria: AOB that convert ammonia to nitrite and nitrite oxidizing bacteria that convert nitrite to nitrate. The study also found that at an initial NH_4_^+^-N concentration of about 66 mg L^−1^, nitrite and nitrate concentrations reached about 18 and 3 mg N L^−1^, respectively, confirming the actual occurrence of nitrification [65]. ABGS can achieve over 90% COD removal, eliminate 80% of total nitrogen through nitrification/denitrification, and uptake up to 95% of phosphorus through polyphosphate accumulating bacteria.

Denitrification and anammox: Within oxygen-limited microzones that develop inside biofilms or aggregates, denitrifying bacteria reduce nitrate and nitrite to gaseous nitrogen (N_2_), achieving complete nitrogen removal. Wang et al. provided direct spatial evidence for this layered microzone structure in an algal–bacterial biofilm system. This layered biofilm provided a suitable environment for the coexistence of multiple microorganisms in complex biofilms, and the final total nitrogen removal efficiency exceeded 90%: algae formed an oxygen-producing zone (0–0.48 mm), AOB consumed oxygen in an oxygen-consuming zone (0.48–0.86 mm), and AnAOB removed nitrogen in an anaerobic zone (>0.86 mm), with *Chlorella* sp., *Nitrosomonas* sp., and *Candidatus Kuenenia* as the dominant algae, AOB, and AnAOB, respectively [66]. Furthermore, anaerobic ammonium-oxidizing bacteria (*Candidatus Brocadia*) contribute to nitrogen removal through the anammox pathway. Chen et al. further demonstrated that coupling anammox with microalgae–bacteria symbiosis successfully integrated nitrification, denitrification, and anammox processes, achieving a maximum TN removal efficiency of 99.51% at an influent NH_4_^+^-N concentration of 100 mg L^−1^, with microalgae promoting the enrichment of denitrifying bacteria and AnAOB. Moreover, in a biofilm system without external carbon or aeration, a peak total nitrogen removal efficiency of 98.8% was achieved through a diversified nitrogen metabolic network encompassing anaerobic ammonium oxidation denitrification, and nitrogen assimilation [67].

The synergistic nature of these pathways is particularly evident in high-ammonia food wastewater treatment. In algal–bacterial biofilm systems, microalgae, ammonia-oxidizing bacteria, and anaerobic ammonium-oxidizing bacteria form an integrated system. Compared to conventional nitrification–denitrification processes, this system operates without additional organic carbon or aeration. However, most studies have been conducted under controlled laboratory conditions; the extent to which these contributions can be manipulated in real food wastewater remains unclear.

## 4. Reactor Configurations for Food Wastewater Treatment

### 4.1. Rotating Algal Biofilm Systems

Rotating algal biofilm systems represent a significant innovation in attached-growth algal–bacterial treatment. A novel vertical rotating microalgae–bacteria symbiotic biofilm reactor was developed to treat mixed wastewater containing municipal and soybean soaking wastewater. This study used a rotating algae biofilm system to treat soy sauce wastewater and achieved the optimal growth rate under a 5-day harvesting cycle. The system analyzed the remediation efficiency and mechanism of rotating algal biofilm (RAB) on high concentration organic wastewater [13]. Liu et al. optimized the rotating algal biofilm system (5-day harvest time, 12 h HRT), achieving a biomass production rate of 3.3 g·m^−2^·d^−1^ and a total ammonia nitrogen removal rate of 82.3% [68]. The developed rotating algal biofilm system (MH-RAB) equipped with mechanical brushes achieves stable operation through 7-day periodic biofilm harvesting. Under the condition of HRT for 48 h, the removal efficiencies of TN and TP reached 90.6 ± 1.8% and 82.8 ± 1.8%, respectively, which were 80.8% and 27.6% higher than traditional RAB systems. Under hydraulic shock (HRT reduced to 8 h) conditions, the removal rates of ammonia nitrogen and nitrite nitrogen remained at 99.0% and 97.3%, respectively. Algae assimilation removes 79.2% of TN and 64.0% of TP [69]. Under sequential batch operation, maximum removal rates for COD, total nitrogen, total phosphorus, and ammonia nitrogen reached 95.6%, 96.1%, 97.6%, and 100%, respectively. During semi-continuous operation, removal rates of COD, TN, and NH_4_^+^–N achieved 98%, 95%, and 99.9%, respectively [3]. The maximum biomass productivity of the biofilm was 2.69 g·m^−2^·d^−1^. Harvested biomass contained approximately 22% carbohydrates, 51% protein, and 10% lipids of the dry weight. This study demonstrated the great potential of the microalgae–bacteria symbiotic biofilm system to treat food and domestic wastewater while harvesting microalgal biomass [3].

The efficacy and mechanisms of rotating algal biofilm systems have also been demonstrated in remediation of soy sauce wastewater. Hu et al. reported that a rotating algal biofilm system achieved superior nutrient removal from soy sauce wastewater—92% COD, 94% ammonium nitrogen, 91% total nitrogen, and 82% phosphorus—with a biofilm productivity of 14 g·m^−2^·d^−1^ under optimized 5-day harvest time and 2-day hydraulic retention time. This high performance was attributed to synergistic interactions within an algae-fungi (Apiotrichum)-bacteria (Acinetobacter and Rhizobia) consortium that assimilated EPS into biomass, and the resulting biomass showed improved protein and essential amino acid contents for potential animal feed applications [13]. The formation of microalgae biofilms depends mainly on the adhesion properties of attachment materials, with optimal attachment materials greatly contributing to wastewater treatment efficiency. Similarly, Gross et al. identified cotton sheet as the best material for algal growth, durability, and cost-effectiveness, and subsequent research demonstrated that nylon and polypropylene mesh with 0.50–1.25 mm openings achieved 28–30 g·m^−2^ biomass yield in a pilot-scale revolving algal biofilm system [70]. Although RAB systems achieve high nutrient removal, their performance varies with harvest cycle, HRT, and carrier material. For instance, the 5-day harvest cycle optimal for soy sauce wastewater may not be transferable to dairy wastewater with different organic loading. Moreover, the energy demand for rotating mechanisms and the risk of biofilm detachment under hydraulic shock remain insufficiently quantified.

### 4.2. Algal–Bacterial Aerobic Granular Sludge

Algal–bacterial aerobic granular sludge (ABGS) represents an advanced configuration that combines the benefits of biofilm and suspended growth systems. ABGS efficiently removes ammonia despite inhibition of nitrifier activity. Elevated specific ammonia removal rates in ABGS indicate enhanced ammonia assimilation under low dissolved oxygen conditions [71]. ABGS offers energy-efficient nitrogen removal in wastewater treatment plants.

Continuous-flow algal–bacterial granular sludge bioreactors with no mechanical aeration have demonstrated excellent performance for recirculating aquaculture wastewater treatment, with daily NH_4_^+^–N removal capacity of 17–31 mg·g^−1^-VSS and >77% of total inorganic nitrogen removal. In this system, the dominant nitrogen removal mechanism shifted from nitrification/denitrification to algal assimilation over the operation period, and 28% of the input nitrogen was recovered in biomass, with dissolved oxygen levels maintained above 9 mg·L^−1^ through photosynthesis and water turbulence without any external mechanical aeration [72]. Similar performance has been reported in a continuous-flow non-aerated microalgal–bacterial granular tubular reactor operated under natural day-night conditions, where over 99% of nitrite-N was removed at a hydraulic retention time of 6 h, and daytime microalgal oxygen production was sufficient to sustain 6-h aerobic conditions during the nighttime [73].

Table 1 summarizes the performance, advantages, and limitations of the main reactor types discussed in this section. Collectively, these findings demonstrate that continuous-flow non-aerated algal–bacterial granular sludge systems offer a promising energy-efficient strategy for recirculating aquaculture wastewater treatment by coupling photosynthetic oxygenation with a shift toward algal assimilation, achieving efficient nitrogen removal and biomass nitrogen recovery without mechanical aeration. Reactor configuration influences not only mass transfer and light availability but also the local cell density that governs QS signaling. For example, attached-growth systems may create localized high-density zones that amplify AHL accumulation, whereas suspended systems may dilute signals. Systematic studies comparing QS activity across reactor types are needed.

## 5. Resource Recovery and High-Value Product Generation

### 5.1. Aquafeed and Animal Feed Applications

The increasing global demand for aquaculture products has intensified pressure on conventional feed resources. Algal–bacterial biomass cultivated on food wastewater offers a sustainable alternative protein source that simultaneously addresses wastewater treatment and feed production.

The protein-rich biomass (up to 51% of dry weight) contains essential amino acids that can partially replace conventional feed ingredients [74]. Integrated microalgal–bacterial cultivation provides a scalable solution for aquaculture wastewater purification with co-produced biomass. Beyond nitrogen removal, algal–bacterial biomass has demonstrated direct nutritional benefits in fish feeding trials. Zhang et al. evaluated ABGS recovered from brackish and marine aquaculture wastewaters as fish feed additives, demonstrating that the harvested biomass contained 33.1–41.3% crude protein with a favorable amino acid profile and was enriched in Mg, K, P, and Fe, confirming its potential as a low-cost substitute for conventional protein-rich feed additives [75]. A composite single-cell protein containing *Chlorella vulgaris* and *Clostridium autoethanogenum* at a 1:4 ratio was evaluated as a fishmeal substitute in largemouth bass (*Micropterus salmoides*) feed, with the FM26 diet (50% fishmeal replacement) yielding the most optimal results: improved disease resistance against *Aeromonas hydrophila*, strengthened antioxidant defense, enhanced immune responses, and increased intestinal digestive enzyme activities [76]. Notably, microalgal co-product biomass from *Nannochloropsis* sp. QH25 has been shown to fully replace fishmeal (100% replacement) in rainbow trout (*Oncorhynchus mykiss*) feeds without compromising fish growth, feed conversion, survival, or fillet composition, while achieving the lowest feed cost per kilogram of fish produced [77]. Furthermore, dietary supplementation with Spirulina (*Arthrospira platensis*) at 50 g kg^−1^ feed in zebrafish resulted in the highest final body weight, weight gain, specific growth rate, and protein efficiency, as well as improved reproductive parameters including gonadosomatic index, eggs per female, fertilization rate, and hatching rate compared to the control group [78]. These findings collectively demonstrate that microalgae and algal–bacterial biomass can serve as effective, sustainable protein sources in aquaculture feeds, contributing to both wastewater resource recovery and aquafeed sustainability. Despite promising feeding trial results, the suitability of wastewater-derived algal–bacterial biomass for aquafeed is constrained by the potential accumulation of heavy metals, pathogens, and pharmaceutical residues. None of the cited feeding trials reported contaminant concentrations in the harvested biomass, leaving a critical data gap for regulatory approval.

### 5.2. Biofertilizers and Soil Amendments

Microalgal–bacterial immobilized co-cultures function as living biofilters for nutrient recovery from wastewater, with demonstrated potential as biofertilizers. For instance, co-immobilized cultures of *Chlorella vulgaris* TISTR 8580 and the plant growth-promoting bacterium *Bacillus subtilis* TISTR 1415, using mixed guar gum/carrageenan binders at a 1:1 ratio, removed 86.7 ± 0.5% of ammonium and 99.3 ± 0.3% of phosphates from synthetic municipal wastewater within 7 days, and the resulting living biofilters were subsequently applied as biofertilizers for 20 days to grow cucumber and sunflower without negative impacts on soil [79].

Algal–bacterial biomass recovered from food wastewater treatment represents a valuable source of biofertilizers and soil amendments. During wastewater treatment, algae and bacteria assimilate nitrogen, phosphorus, potassium, and micronutrients, concentrating these nutrients into harvestable biomass. For example, an algal–bacterial consortium treating food waste liquefied liquid achieved 65.88–97.69% purification through nutrient assimilation, demonstrating the direct coupling of food waste treatment with biofertilizer production. Wastewater-derived algal biomass has also been shown to exert positive effects on crop growth when applied as a soil amendment, suggesting that its agronomic value may extend beyond simple fertilization to include broader biostimulatory functions [80]. The slow-release characteristic of algal biomass is one of its most significant advantages as a fertilizer. Nutrients within algal biomass are primarily bound within organic cellular structures and require microbial mineralization before becoming fully available to plants, reducing nutrient losses through leaching and volatilization [81].

### 5.3. Biofuels and Bioenergy

Co-cultures of microalgae with bacteria have shown promise not only in wastewater treatment but also in the production of valuable bioproducts such as biofuels [13]. The lipid and carbohydrate content of algal–bacterial biomass makes it suitable for biofuel production. QS molecules in wastewater have been shown to trigger microalgae lipid synthesis [82]. With QS stress, the lipid content of *Chlorophyta* sp. increased by approximately 84%, while biomass production decreased only slightly. Lipid productivity and production improved by 88% and 86%, respectively, with enzymes on the fatty acid synthesis pathways generally upregulated [82]. Li et al. further demonstrated that all three AHLs (C4-HSL, C6-HSL, and 3-oxo-C10-HSL) enhanced total nitrogen and total phosphorus removal and stimulated biomass growth while redirecting microalgal carbon allocation toward lipid accumulation in dialysis membrane–algal–bacterial photobioreactors [35]. Co-culture of *Chlorella* and *Scenedesmus* could enhance total lipid production under bacteria QS molecule stress, with lipid production in the co-culture system 20–79% higher than monoculture. The maximum lipid productivity and production of the co-culture system were 0.12 g L^−1^ d^−1^ and 0.71 g L^−1^, respectively, and cells in the co-culture system counteracted oxidative stress induced by AHLs [83]. These findings collectively indicate that QS and algal–bacterial co-culturing synergistically enhance microalgal lipid accumulation, enabling simultaneous wastewater treatment and biofuel production.

### 5.4. Bioactive Compounds and Nutraceuticals

Co-culture of microalgae and bacteria has emerged as an effective strategy for the production of bioactive compounds with antimicrobial, antioxidant, and anti-inflammatory activities [84]. Microalgae are rich sources of polyunsaturated fatty acids (20–50% of total lipids), carotenoids such as astaxanthin and β-carotene (up to 3% of dry weight), peptides, and sulfated polysaccharides (5–20%), all exhibiting antioxidant, antimicrobial, anti-inflammatory, and anticancer activities [84,85]. When cultivated in co-culture with bacteria, microalgae undergo metabolic changes that lead to the production of novel compounds or an increase in the yield of known metabolites [84]. Notably, chemical extracts from high-biomass algal–bacterial co-culture systems have demonstrated bioactivity against *Bacillus subtilis* and *Candida albicans*, with 23 extracts showing antimicrobial activity [86]. Co-cultivation of *Chlorella minutissima* with bacteria achieved 45 mg gallic acid equivalents g^−1^ phenolics, 12 mg g^−1^ carotenoids, 75% DPPH radical scavenging, and 18 mm antifungal zones against *Candida albicans* [87].

Table 2 compares the main biomass valorization pathways with respect to feedstock composition, processing route, potential benefits, technology maturity, and implementation challenges. The ecological interactions between microalgae and bacteria, mediated by nutrient exchange and chemical signals such as QS, promote the synthesis of bioactive compounds, and environmental factors such as pH, temperature, light intensity, and cell density directly influence production efficiency [84]. Microalgal consortia, particularly microalgae–bacteria consortia, have been identified as suitable systems for enhancing the production of biomolecules with anticancer, antioxidant, anti-aging, antidiabetic, and anti-inflammatory potential [85]. Among the pathways compared, aquafeed and biofertilizer applications are supported by pilot-scale feeding and field trials, but their deployment is constrained by contaminant accumulation and the absence of standardized quality criteria. Biofuel production from algal–bacterial biomass remains largely at laboratory to pilot scale, with downstream extraction energy demand as the main economic barrier. Bioactive compounds show high potential value but are limited by low intracellular content and the cost of extraction and purification. Across all pathways, technology maturity is constrained by the lack of long-term, full-scale data and by regulatory uncertainty regarding wastewater-derived biomass. These differences should be considered when selecting valorization routes for a given wastewater source and product market.

### 5.5. Extracellular Polymeric Substances as Bioflocculants and Hydrogels

In algal–bacterial wastewater treatment, extracellular polymeric substances (EPS) are considered predominantly as structural constituents of the biofilm matrix and as a process constraint that exacerbates membrane fouling and complicates dewatering, yet they also represent a recoverable high-value product whose carboxyl, hydroxyl, and amino groups provide the adsorption sites and bridging that underlies flocculation [42,45,46]. EPS from Scenedesmus acuminatus dosed back to the same culture at 3.2 mg g^−1^ lowered the alum requirement from 77.6 to 4.5 mg g^−1^ of biomass and the chemical cost of harvesting from approximately USD 282 to USD 71 per metric ton of dry biomass [88]. Because no metal coagulant is added, the biomass remains suitable for food, feed, and fertilizer uses, consistent with the contaminant constraints of Section 6; flocculation is nevertheless pH-dependent and most efficient near the isoelectric point [89].

Structural EPS and alginate-like extracellular polymers (ALE) from biological sludge form hydrogels through ionic cross-linking with Ca^2+^ and carry functional groups approximately 60% similar to those of commercial alginate [89]. ALE recovery from algal–bacterial granular sludge can be coupled with phosphorus recovery and increases with the organic loading rate, reaching approximately 13.4 mg g^−1^ of volatile suspended solids [90]. The resulting hydrogels retain up to 99.5 wt% of water and, after loading by swelling in KNO_3_ solution, release up to 133 mg K^+^ and 187 mg NO_3_^−^ per gram of total solids [91], making them candidate soil conditioners and slow-release fertilizer carriers. Two limitations should be acknowledged. Extraction requires thermo-alkaline reagents and appreciable energy, and the recovered polymer retains part of the wastewater contaminant load, which may require post-treatment for food- and feed-grade use.

## 6. Challenges and Limitations

The high cost of biomass production and downstream processing remains a primary barrier to commercialization. Microalgae cultivation in open systems offers lower capital costs but faces contamination risks and variable productivity, whereas closed photobioreactors provide greater control at substantially increased capital investment and operational costs. Downstream processing, including harvesting, dewatering, drying, and extraction, represents a major cost driver, and the high moisture content of harvested biomass makes drying one of the most energy-intensive stages. These economic constraints collectively limit the competitiveness of algal–bacterial products against conventional alternatives [92,93].

Beyond economic constraints, contaminant accumulation and safety concerns pose significant challenges. Wastewater-grown algal biomass may accumulate heavy metals, pathogens, pharmaceuticals, and other contaminants present in the wastewater, which is particularly problematic for food, feed, and agricultural applications where contaminant concentrations are strictly regulated [94]. The potential for contaminant transfer through the food chain is a major concern, and risk mitigation requires careful selection of wastewater sources, routine monitoring of biomass quality, compliance with feed safety standards, and appropriate pretreatment methods.

Finally, the complex microbial interactions within consortia, including metabolic cross-feeding, signaling interactions, and community dynamics, remain incompletely understood, and current understanding of how operational parameters influence consortium structure and function is limited [95,96]. In addition, long-term safety data for wastewater-derived algal products are scarce, as most studies have focused on short-term laboratory experiments with limited data on the effects of prolonged consumption of algal biomass containing trace contaminants.

Beyond these process-level constraints, several barriers determine whether algal–bacterial systems can be implemented at full scale. Productivity in open ponds fluctuates seasonally because light and temperature drive photosynthesis: in a life-cycle assessment of a 10,000 population-equivalent (p.e.) pond system, areal biomass production fell from 25.8 g TSS m^−2^ d^−1^ in summer to 3.3 g TSS m^−2^ d^−1^ in winter, and the hydraulic retention time had to be more than doubled in the cold season to maintain effluent quality [97]; comparable seasonal swings occur at full scale [98], where solar radiation and influent ammonium concentration, rather than operator-controlled set-points, dominate long-term productivity in high-rate algal ponds [99]. Design must therefore be based on the least favourable season, which further penalizes the already large footprint and capital cost, and land availability is itself a structural constraint: area requirements of about 4 m^2^ per p.e. for a 10,000 p.e. system [97] are several-fold larger than those of conventional activated sludge and restrict deployment to locations where land is available and inexpensive [97,99]. Downstream, suspended cultures are dilute, typically containing less than 1 g L^−1^ of dry solids, so that harvesting and dewatering dominate both the energy demand and the capital expenditure of the process [100]; attached-growth systems concentrate the biomass on a carrier, spool harvesting yielding 12–16% solids [101] and an enhanced rotating algal biofilm reactor about 38 g m^−2^ d^−1^ at pilot scale after scale-up to 20,000 m^3^ d^−1^ [102], but they perform only within a narrow window of biofilm thickness and shear, since thick biofilms limit light and substrate penetration while weak adhesion between cells and the carrier causes sloughing that reduces biomass recovery and degrades effluent quality [103], and controlled detachment still relies on labour-intensive mechanical devices that are difficult to scale [101]. Where membranes are used for biomass retention, thickening or polishing, fouling by algal–bacterial cells and their extracellular polymeric substances remains the principal limitation, requiring frequent cleaning and periodic membrane replacement and shifting the cost burden towards energy and maintenance [100]. Finally, the regulatory and evidentiary framework lags behind the technology: Regulation (EU) 2020/741 harmonizes the quality of reclaimed water but not the status of the algal biomass generated in the process, whose use in food and feed requires a case-by-case safety assessment in the absence of harmonized end-of-waste criteria and of validated monitoring protocols for trace contaminants and antibiotic resistance genes [104]; and because most published results still come from laboratory and small pilot reactors operated over weeks or months, microalgae-based wastewater treatment remains at technology readiness levels of approximately 4–6 despite more than six decades of research [102], with techno-economic and life-cycle assessments based largely on extrapolated design assumptions rather than on measured data from operating plants [97,102,105]. Generating long-term, full-scale datasets covering multiple seasons, contaminant accumulation and process stability should therefore be a priority for future research [98,99].

## 7. Future Directions and Emerging Opportunities

Future research should prioritize the elucidation of QS mechanisms in algal–bacterial symbiosis systems. The variations in AHLs provide direct evidence for interspecies communication, facilitating ecological reconfiguration in biofilms. However, the specific signaling pathways, receptor mechanisms, and downstream metabolic effects of QS in different algal–bacterial pairs remain poorly understood. Research should investigate how QS signals coordinate carbon and nitrogen metabolism, regulate EPS composition, and modulate stress responses under food wastewater conditions. The potential for engineering QS pathways to enhance desirable system properties, including nutrient removal, biomass productivity, and target product accumulation, represents an exciting frontier. In parallel, the development of next-generation strains with improved productivity, stress tolerance, and processing characteristics represents a critical research priority. Strain selection should consider not only nutrient removal efficiency but also biomass composition, contaminant tolerance, and harvestability. Synthetic biology and metabolic engineering offer opportunities to enhance desirable characteristics; by promoting up-regulation of gene expression in bio-augmentation bacteria, QS enhances their colonization rates and improves contaminant degradation. However, the commercialization of engineered strains requires robust safety assessment frameworks.

Process optimization and artificial intelligence are emerging as promising tools for analyzing operational datasets and supporting predictive process control. These technologies can optimize cultivation parameters, predict system performance under varying conditions, and support real-time process control. The optimization of reactor design and operational parameters remains a priority; research should focus on achieving stable long-term performance in open and semi-open cultivation systems, addressing challenges including biofilm detachment, self-shading, grazing pressure, and shifts in microbial community composition. The integration of algal–bacterial wastewater treatment with downstream biorefineries offers opportunities to maximize resource recovery and economic viability [106,107]. Cascading resource recovery, in which multiple products are sequentially extracted from the biomass, can improve overall biomass utilization. The circular economy model achieves a win–win for environmental and economic benefits, and integrating algal cultivation with wastewater treatment and CO_2_ fixation enables a closed-loop model that closely links wastewater, microalgae, food, and byproduct utilization. Finally, comprehensive techno-economic analysis (TEA) and life-cycle assessment (LCA) should be integrated from the early stages of process development to identify cost drivers, environmental hotspots, and opportunities for optimization. Regional variations in climate, wastewater composition, energy supply, infrastructure, and potential markets for algae-derived products should be considered.

## 8. Conclusions

Algal–bacterial symbiotic systems represent a promising circular-economy platform for treating ammonia-rich food wastewater while simultaneously recovering nutrients and generating high-value products. This review has synthesized the mechanistic foundations, reactor configurations, resource recovery pathways, and commercialization barriers associated with these systems. The complementary metabolic activities of microalgae and heterotrophic bacteria are mediated by oxygen and carbon dioxide exchange, nutrient cross-feeding, extracellular polymeric substance dynamics, and QS. These interactions create self-sustaining treatment systems. Such systems reduce external aeration requirements and produce harvestable biomass enriched in proteins, lipids, carbohydrates, and bioactive compounds. QS, particularly through AHL-mediated signaling, is one of several regulatory mechanisms that modulate microbial community succession, biofilm formation, EPS production, metabolic pathway expression, and stress tolerance. Together with gas exchange, nutrient assimilation, and microbial metabolism, it contributes to pollutant removal efficiency and biomass quality. Nitrogen removal in these systems proceeds through multiple coordinated pathways, including coupled nitrification and denitrification, algal assimilation, and anaerobic ammonium oxidation. Among these pathways, algal assimilation plays a quantitatively dominant role under low dissolved oxygen conditions. Recent advances in reactor configurations, including rotating algal biofilm systems and algal–bacterial granular sludge, have demonstrated scalable performance with high nitrogen removal efficiencies and simultaneous biomass production. The harvested biomass offers versatile valorization opportunities, including aquafeed, biofertilizers, biofuels, and bioactive compounds with antimicrobial, antioxidant, and anti-inflammatory activities.

Despite these advances, several interconnected challenges continue to hinder large-scale implementation. The high cost of biomass production and downstream processing, variability in biomass quality, concerns regarding contaminant accumulation in wastewater-grown biomass, and incomplete understanding of complex microbial interactions within consortia all pose significant barriers. Addressing these challenges will require coordinated efforts in several directions: elucidating the specific signaling pathways, receptor mechanisms, and downstream metabolic effects of QS in different algal–bacterial pairs; developing next-generation strains with improved productivity, stress tolerance, and processing characteristics through synthetic biology and metabolic engineering; integrating artificial intelligence and machine learning for predictive process control and reactor optimization; advancing integrated biorefinery concepts that maximize cascading resource recovery; and embedding comprehensive TEA and LCA from the early stages of process development. Three priorities follow for industrial implementation: establishing long-term demonstration-scale performance data under real food wastewater conditions, standardizing biomass quality and safety assessment, and integrating techno-economic analysis and life-cycle assessment from the early stages of process design. Progress on these priorities is more likely to determine whether algal–bacterial systems move from laboratory research to practical application. By bridging fundamental mechanistic understanding with applied engineering solutions, algal–bacterial symbiosis system can transition from laboratory research toward practical food wastewater treatment and resource recovery, contributing to a more sustainable and circular food supply chain.

## Figures and Tables

**Table 1:** Comparison of reactor configurations for algal–bacterial treatment of food wastewater.

Reactor Type	Growth Mode	Conditions	Operational Requirements	Biomass Productivity	Advantages	Limitations	Reference
Vertical rotating algal–bacterial biofilm	Attached growth	Municipal + soybean soaking wastewater; sequencing batch/semi-continuous	HRT: not specified; harvesting: periodic; aeration: none	2.69 g m^−2^ d^−1^	Simultaneous N and P removal; biomass with 51% protein	Light and mixing uniformity challenges during scale-up	[3]
Rotating algal biofilm (MH-RAB)	Attached growth	Mechanical brushes; 7-day harvest, HRT 48 h	HRT 48 h; harvesting: 7-day cycle; aeration: none	—	High shock resistance; high algal assimilation contribution	Brush maintenance; long-term stability needs validation	[67]
Rotating algal biofilm (RAB)	Attached growth	Soy sauce wastewater; 5-day harvest, 2-day HRT	HRT 48 h; harvesting: 5-day cycle; aeration: none	14 g m^−2^ d^−1^	Simple harvesting; high protein content; resistance to hydraulic shock	Energy demand for rotation; carrier material affects attachment; light penetration limited at scale	[1]
Algal–bacterial granular sludge (ABGS)	Granular/suspended	Continuous flow, no aeration, aquaculture wastewater	HRT: not specified; harvesting: settling; aeration: none	28% of input N recovered in biomass	No mechanical aeration; energy-efficient; stable granules	Long start-up period; susceptible to water quality fluctuations	[71]
Continuous-flow non-aerated ABGS tubular reactor	Granular/suspended	Natural day–night cycle, HRT 6 h	HRT 6 h; harvesting: settling; aeration: none	—	Daytime O_2_ production sustains nighttime aerobic conditions; energy-saving	Limited scale-up and long-term operation data	[72]

**Table 2:** Summary of biomass valorization pathways from algal–bacterial systems treating food wastewater.

Valorization Pathway	Feedstock /Biomass Composition	Key Processing	Potential Benefits	Technology Maturity	Main Challenges	References
Aquafeed	Protein (up to 51% dry weight), essential amino acids, Mg, K, P, Fe	Drying, cell disruption, feed formulation	Replaces fishmeal; improves growth, immunity, and disease resistance	Pilot to early commercial (feeding trials in multiple fish species)	Accumulation of heavy metals, pathogens, and pharmaceutical residues; lack of contaminant data; regulatory restrictions	[74,75,76,77,78]
Biofertilizer	N, P, K, micronutrients, organic carbon	Direct application, composting, slow-release formulation	Slow nutrient release; improved soil structure; enhanced crop growth	Pilot scale (field trials limited)	High moisture content requires drying; contaminant risks; difficulty in product standardization	[79,80,81]
Biofuel	Lipids (up to 48%), carbohydrates	Transesterification to biodiesel; fermentation to ethanol; anaerobic digestion to methane; dark fermentation to hydrogen	Renewable energy; utilizes lipid and carbohydrate fractions	Laboratory to pilot scale	High production cost; energy-intensive downstream extraction; scale-up challenges	[82,83,84,85]
Bioactive compounds	Carotenoids (astaxanthin, β-carotene), polysaccharides, peptides, PUFAs	Solvent extraction, supercritical fluid extraction, enzyme-assisted extraction	Antioxidant, antimicrobial, anti-inflammatory, anticancer activities	Laboratory to early pilot scale	Low content; high extraction cost; lack of long-term safety data; regulatory inconsistency	[84,85,86,87]

## Data Availability

No new data were created or analyzed in this study. Data sharing is not applicable to this article.
